# Gallstones: A Worldwide Multifaceted Disease and Its Correlations with Gallbladder Carcinoma

**DOI:** 10.1371/journal.pone.0166351

**Published:** 2016-11-10

**Authors:** Raj Kumar Sharma, Kanchan Sonkar, Neeraj Sinha, Pradeep Rebala, Ahmad Ebrah Albani, Anu Behari, Duvvuri Nageshwar Reddy, Alvina Farooqui, Vinay Kumar Kapoor

**Affiliations:** 1 Centre of Biomedical Research, Sanjay Gandhi Post Graduate Institute of Medical Sciences (SGPGIMS) - Campus, Lucknow, India; 2 Department of Biosciences, Integral University, Kursi road, Lucknow, India; 3 Department of Biochemistry & BiophysicsUniversity of Pennsylvania Perelman School of Medicine, Philadelphia, United States of America; 4 Department of Surgical Gastroenterology, Asian Institute of Gastroenterology, Somajiguda, Hyderabad, Andhra Pradesh, India; 5 Department of Surgery, Zayad Military Hospital, Abu Dhabi, UAE; 6 Department of Surgical Gastroenterology, SGPGIMS, Raibarelly Road Lucknow, Uttar Pradesh, India; National Cheng Kung University, TAIWAN

## Abstract

**Background:**

Gallstones (GS) associated diseases are among the most recurrent and frequent diseases delineated in India and United Arab Emirates. Several reports suggest that the association of GS with gallbladder cancer (GBC) is very high in Northern part of India; however, its occurrence in UAE and Southern part of India is notably low. Therefore, in the present study, we aimed to perform compositional analysis of GS in three different geographical areas by Solid State Nuclear Magnetic Resonance and Fourier Transformed Infrared spectroscopy.

**Methods:**

Natural abundance ^13^C cross polarization magic angle spinning Nuclear Magnetic Resonance and Fourier Transform Infrared spectroscopy is employed for the analysis of human gallstone.

**Results:**

Cholesterol, bilirubin and calcium carbonate were present in variant concentrations in GS obtained from three different geographical regions. Cholesterol was present predominantly in gallstones from North India. Bilirubin was found to be a main constituent in gallstones pertaining to South India. Whereas GS from UAE showed both cholesterol and bilirubin as their major constituents. Calcium carbonate was found in varying concentrations in gallstones acquired from different regions.

**Conclusion:**

Variation in environmental condition and dietary habits may contribute and affect the GS formation. Alterations in bile composition influence the GB and augment the crystallization of cholesterol. Analysis of different geographical regions GS could be an important stride to understand the etiology of GS diseases.

## Introduction

Gallstone (GS) disease is a very common health problem that affects millions throughout the world [1, 2]. GS are generally found in gallbladder (GB) which occupies the central part of human biliary system. GB acts as a reservoir of the bile fluid produced by the liver. Bile is concentrated in GB and drained out for lipid absorption. GB epithelium is most absorptive epithelium surface which facilitates bile concentration in GB by trans-mucosal absorption. Bile constitutes cholesterol, bilirubin, bile salts and phospholipids which are the key components to keep up the homeostasis of GB. There are various pathological conditions that can disturb the regulation of these secretions. Altered secretion of biliary cholesterol, phospholipids and bile acids disturbs the solubility of bile [3]. GS formation commences with cholesterol super saturation [4]. This corresponds to excess amount of cholesterol secretion or owing to less concentration of bile salts and phospholipids which are necessary for solubility of cholesterol [5, 6]. Secretion of cholesterol increases with age and correlated with dietary factors and fasting state. Cholesterol forms crystals that remains in the gall bladder and combines with other constituents like calcium salt, magnesium salts and bilirubin to form GS. GS occurs in various shapes (round, angular, oval and so on), sizes (from few millimeters to 6 cm), color (creamy white, yellow, black and brown) and can be amorphous or crystalline [7]. Worldwide distribution of GS has been described in several reports. There is notorious relationship with GS and GBC. Patients having history of GS are more susceptible to GBC. The most important risk factor for developing GBC is attributed to long standing period and GS size measuring greater than 3.0 cm [8]. Nevertheless, there is no proven evidence of association between GS and GBC.

In accordance with the above mentioned context the composition of GS obtained from different environmental conditions and geographical regions may offer clues to the pathogenesis of GBC, therefore determining the composition of GS becomes very important. There are various techniques that can be employed for this purpose. These techniques include X-ray diffraction, fluorescent spectroscopy, Fourier transform infrared spectroscopy (FTIR) and Nuclear Magnetic Resonance (NMR) Spectroscopy. These techniques have contributed meaningful insight and information.

NMR spectroscopy has applicability to analyze biological samples in disease conditions with minimal sample preparation and reproducibility. Both solid and solution form of samples can be analyzed by NMR spectroscopy. For using NMR in solution state, sample is dissolved in a suitable solvent and for solid state; sample can be used as powder, solid or crystalline form. ^13^C CP-MAS provides complete structure analysis of organic molecule as it takes into consideration of all organic constituents in GS both soluble and non-soluble. FTIR spectroscopy has been used as an important technique for characterizing biomolecules. Quick results make it an excellent technique. FTIR is important to obtain information about organic and inorganic constituents of GS. It gives clear absorption peaks for organic and inorganic constituents.

In the present article we report analysis of GS from North India, South India and UAE which are geographically very different. For this purpose we have used Solid-State NMR in combination with FTIR spectroscopy to characterize human GS from three different geographical regions: first with very high prevalence rate of GS disease as well as high incidence rate of GBC- North India, second with high prevalence rate of GS disease but low incidence rate of GBC- United Arab Emirates (UAE) and third with low rates of both GS disease and GBC- South India.

## Material and Methods

### Study samples

In the present study, human GS from three different geographical regions, North India (specifically Lucknow, Uttar Pradesh), South India (specifically Hyderabad, Andhra Pradesh) and United Arab Emirates (UAE) were included. These GS were collected from 134 patients undergoing cholecystectomy for cholelithiasis only; no stones from GBC patients were included. GS were collected from Department of Surgical Gastroenterology, Sanjay Gandhi Post Graduate Institute of Medical Sciences(SGPGIMS), Lucknow in North India (n = 67, age mean 47), Zayed Military Hospital, Abu Dhabi, UAE (n = 56, age mean = 45-years) and Asian Institute of Gastroenterology, Hyderabad in South India (n = 11, age mean = 46-years). After removal, GS were washed with double distilled deionized water to remove all the traces of bile and were stored in airtight vials at room temperature until experiments were performed. For fair comparison, GS from patients used in the study were age and sex matched. In cases where multiple stones were present, only one stone was used for analysis. Study protocol was approved by institutional Ethical Committee (SGPGIMS). Informed consent in written form was obtained from all the participants.

### Experimentation

#### Solid state NMR experiments

The ^13^C CPMAS experiments of cholesterol crystals, bilirubin and GS samples were performed at room temperature. All NMR spectra were recorded on Bruker Biospin Avance 600 MHz spectrometer (Avance III, Bruker Biospin, Switzerland), operating at 600.15 MHz for proton ^1^H resonance frequency and 150.15 MHz for carbon ^13^C resonance frequency using Bruker 3.2 mm Magic Angle Spinning (MAS) E-Free probe. Prior to NMR experiments, GS were finely grounded with the help of pestle and mortar and around ~25 mg of GS was used to fill the 3.2 mm Zirconia rotor. ^13^C CPMAS spectra were then recorded with the following parameters: Magic angle spinning (MAS) frequency of 5.0 KHz, contact time 1.0 ms, 512 scans, RG value 90.5, spectral width 315 ppm, 2 k data points and 10 sec recycle delay. All spectra were processed with 20 Hz line broadening. ^13^C CPMAS spectra of standard cholesterol crystals and bilirubin were also recorded accordingly.

#### FTIR spectroscopy

FTIR spectroscopy was also performed for the GS analysis. FTIR spectra for all samples were recorded using potassium bromide (KBr) pellet method. For preparation of these pellets, reference and GS samples were mechanically grounded in very fine powder form. These powdered samples were then thoroughly and carefully mixed with KBr by hand using pestle mortar with 2:98 (w/w) samples to KBr ratio. FTIR spectra were recorded using Perkin-Elmer Spectrometer. Spectra were acquired in the mid-infrared region (4000 to 400 cm^-1^) at a resolution of 2 cm^-1^. 16 scans were accumulated for each spectrum with overall acquisition time of 2 min. Absorbance was recorded with respect to blank or control sample.

#### Statistical analysis

A total of 115 GS sample from different regions were considered for statistical analysis. To find significant variation of cholesterol determined by NMR among groups. We have performed one-way analysis of variance (ANOVA) with Boneferroni correction and Mann-Whitney U-test under non-parametric method. Analysis was performed by SPSS (version 11.2, IBM USA) software.

## Results and Discussion

The present study implies to investigate the prevalence of GS disease among the population inhabiting different geographical areas. Understanding the pathogenesis of gallbladder cancer is very important in order to perform appropriate disease management so that mortality rate can be minimized. In this direction, composition analysis of GS can provide important missing links between these two. Analysis of GS from three different geographical regions; first with high prevalence rate of both GS disease and GBC (North India), second with high prevalence rate of GS disease but low prevalence rate of GBC (UAE) and third with low prevalence rates of both GS disease and GBC (South India) has been performed to see the differences in their composition. Two different spectroscopic techniques, solid state NMR spectroscopy and FTIR spectroscopy were used for the analysis.

### Earlier studies performed

Analyzing GS is very important to know the etiology of disease, to interpret the factors behind its formation and association with GBC. Several studies have been performed for analyzing GS. A similar study based on GS analysis and characterization was performed using NMR, FTIR, XRD and SEM and involved patients from England and Germany. The study showed that all GS comprises cholesterol monohydrate and calcium along with Fe, Ni, Pb, and Cu elements. In the same study concentration of Zn and Mn were found to be higher in GS from England [9]. Bile salts bind with metals which plays an important role in GS formation. Another study performed on GBC samples obtained from Indian and Japanese patients showed that the heavy metal exposure may increase the risk of GBC, and also reported elevated level of Cr, Pb, Ar and Zn [10]. Elevated level of cholesterol, Ca and Mg in GS was also reported in NMR based study performed by Srivastava et al [11]. In yet another, analysis by NMR have characterized that cholesterol polymorphs was found to be different in different GBC samples [12]. One study executed by FTIR, found mainly cholesterol and bilirubin as major component in GS samples. They have also found bilirubin as one of the important constituents of black stones. Study on GS by ICP-MS, showed the role of metals Fe, Pb, Cd, Ni in GS formation [9]. Another study on GS from South western Finland showed the presence of anhydrous form of cholesterol as main constituents in combination with calcium carbonate. These stones also contain apatite, calcium palmitate, and sodium chloride in lesser amounts [13]. In yet another study using X-ray diffraction technique, which confirmed the presence of the monohydrate as well as anhydrous polymorphs of cholesterol. This study also gave the notion that, there may be other polymorphs of cholesterol which can also be present in GS. This study also revealed that the composition of stones changes from periphery to center [14].

### Morphological features of GS

**Fig 1:**
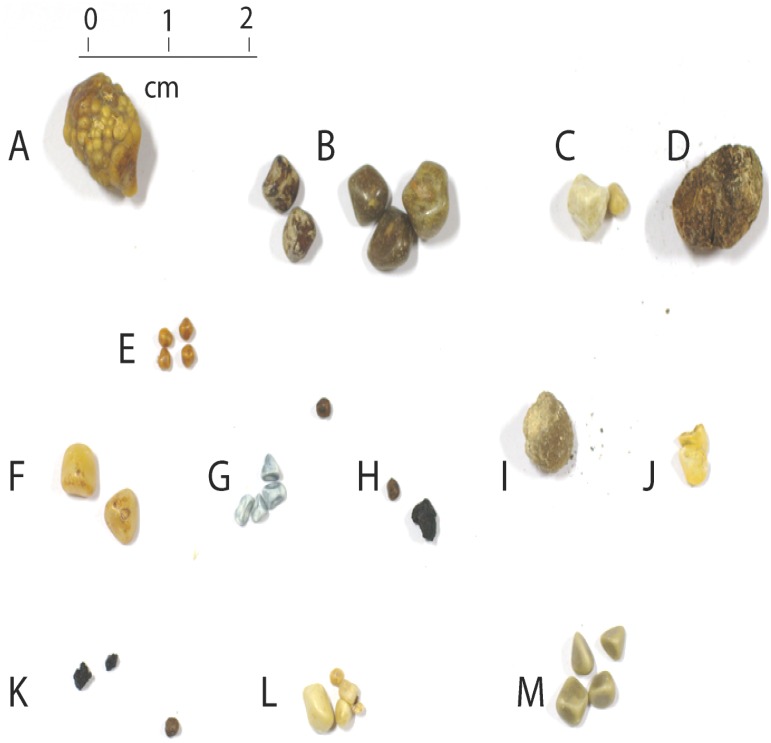
Morphological features of human Gallstones.

Geographically different GS were included in this study. We have found varieties of GS in our analysis. There are no definite characteristics stipulating the morphological features of GS. GS may differ by color, shape and size. GS from different regions cannot be differentiated on the basis of geographical region either by color, size or shape. In all regions single, double and multiple GS were found (Fig 1). Predominantly there are three type of GS, cholesterol GS, bilirubin GS and mixed GS. Cholesterol GS and mixed GS are primarily brown, dark brown and brownish yellow in color. Whereas pigmented GS comprise black color. In our analysis we have found cholesterol and mixed GS color pattern for North India and UAE GS. GS from both regions were rich yellow, brownish yellow but few of them were black, while maximum GS from South India were black in color.

#### GS size

GS were considerably different on the basis of size. Most of the GS from North India were relatively bigger in size (1–3 cm) as compared to those from UAE (5 mm- 1.5 cm) and from South India (1 mm– 1 cm).

#### GS color

Yellow, yellowish brown, brownish black, dark brown and black stones were found more frequent. Many white and grey stones were also present. Black color was dominant in South Indian GS.

#### GS shape

GS were generally round, spheroid to oval shaped. Outer surface was also not similar in all GS. Around 50% GS from South India were very hard and showed ceramic nature, whereas only 8% of GS from UAE showed this feature, none of the GS from North India was hard.

### NMR studies

Nuclear magnetic resonance (NMR) is emerging as an important diagnostic tool for many different diseases, as it is rapid, non-invasive and highly reproducible, requiring small amount of sample and a single experiment provides much information on all the constituents/ molecules present in a sample without any ambiguity [15]. Due to highly specific and quantitative nature of NMR, various components of GS can be easily identified. Quantitative information can be achieved by comparing peak intensity with respect to references of known concentration. Polymorphism of cholesterol in GS will be reflected in the ^13^CPMAS; as different polymorphs of cholesterol have their own characteristic spectral patterns [12]. NMR spectroscopy has also been employed for studying GS using both solution and solid state techniques. Solution state NMR study on GS also showed cholesterol as major constituent with calcium and magnesium salts [11]. Statistically elevated amount of calcium and magnesium was found in malignant GS disease whereas cholesterol concentration was statistically reduced in malignant cases. Solid state NMR study showed the presence of only cholesterol as organic component. This study also confirmed the presence of different polymorphs of cholesterol in GS [12].

### Qualitative ^13^C solid state NMR results

**Fig 2:**
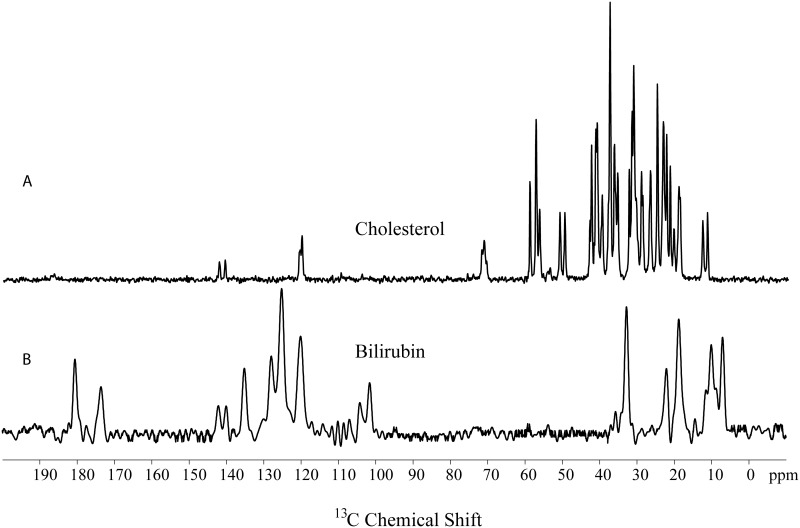
Solid-state NMR spectra of reference compounds (A) ^13^C CPMAS spectra of re-crystallized cholesterol crystal and (B) bilirubin.

**Fig 3:**
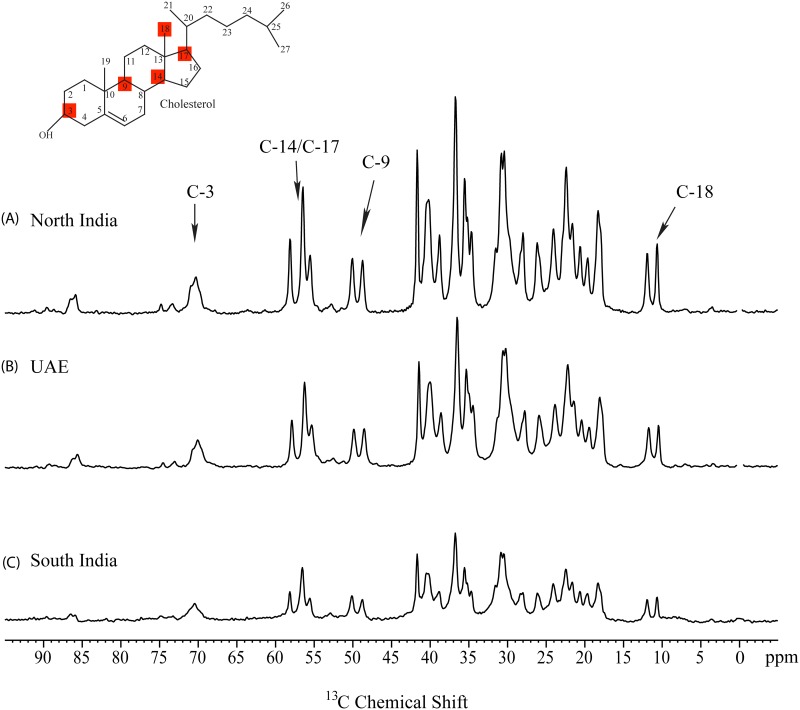
^13^C CPMAS NMR spectra of GS from the regions with, (A) high prevalence rates of both GS disease and GBC (North India) showing presence of cholesterol, (B) high prevalence rate of GS disease but low prevalence rate of GBC (UAE) and (C) low prevalence rate of both GS disease and GBC (South India) and representative peaks used for the identification of cholesterol were C-18 (11.0 ppm), C-9 (50 ppm), C-14/C-17 (57 ppm) and (70 ppm).

**Fig 4:**
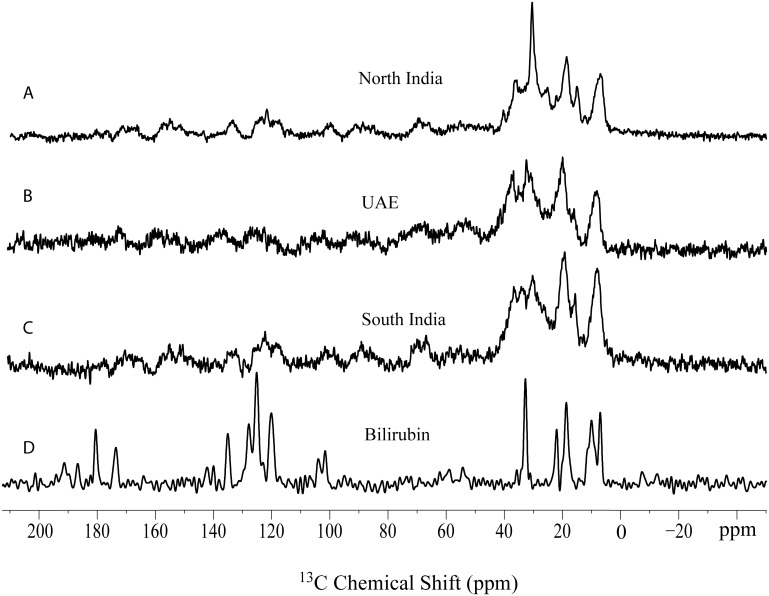
^13^C CPMAS NMR spectra of gallstones from three geographical regions (A) North India, (B) UAE and (C) South India showing the presence of bilirubin in them. For comparison, ^13^C CPMAS NMR bilirubin spectrum (D) is also incorporated.

We have recorded ^13^C NMR spectra of standard cholesterol crystals and bilirubin (Fig 2). Stack plot of GS spectra from three regions along with cholesterol structure and assignment of representative peaks are given in (Fig 3). This shows the presence of cholesterol as the main organic component. However, there are few stones which showed broad signals. Only 2 out of 67 GS from North India showed broad signals instead of sharp signal of cholesterol similar to stones from UAE and South India indicating the absence of organic components in these stones (Fig 4A). Morphologically these two GS were black in color, very small sized and were hard as compared to other GS from North India.^13^C NMR spectra from other 65 GS showed the presence of only one type polymorphic cholesterol crystal. Their spectral pattern showed that they were monohydrate and amorphous type of cholesterol crystal. None of the 67 gallstone showed the anhydrous form of cholesterol crystal, Representative peaks used for the identification of cholesterol were C-18 (11.0 ppm), C-9 (50 ppm), C-14/17(57 ppm) and C-3 (70 ppm).

GS from UAE showed the presence of cholesterol as main organic component. Most of GS were cholesterol GS as cholesterol constituted more than 50% of their weight. Cholesterol polymorphs present in these GS were of two types, one type was similar to those present in GS from North India and South India that is i.e. monohydrate with amorphous type of cholesterol crystal as evident by their C-18 chemical shift whereas only one GS showed the presence of anhydrous form of cholesterol crystal. This anhydrous crystal type was identified by C-18 chemical shifts (11.7 ppm, 12.7 ppm, 13.0 ppm and 13.8 ppm) and specific spectral pattern of anhydrous form of cholesterol crystal. 12 out of 56 GS showed the presence of broad signals (Fig 4B) instead of sharp signals of cholesterol. Upon careful examination, it was clear that these broad signals were showing pattern similar to those of ^13^C NMR spectra of bilirubin but their broad resonance indicated the presence of some metallic component nearby which could not be identified. Morphologically these GS were harder, smaller and black in color. ^13^C NMR spectra of GS from South India also showed the presence of cholesterol as the main organic component. 6 out of 11 GS showed the presence of cholesterol as their sole organic component, but 5 of the 11 GS showed the presence of very broad signals (Fig 4C) Similar to those present in the twelve UAE stones. Their broad signals pattern resembled with that of bilirubin, but it also suggested the presence of metallic component in close proximity of organic component bilirubin. These 6 GS were very small in size as well as were black and very hard whereas other GS were soft in nature and were bigger and they were of different colors. GS spectra which showed the presence of cholesterol were similar to those from North India, indicating the presence of single polymorph i.e. that is cholesterol monohydrate with amorphous crystals. All characteristic resonances and spectral pattern were clearly detectable. From ^13^C NMR analysis, it is clear that GS from these three regions have cholesterol as their main organic component. By comparing ^13^C NMR spectra of bilirubin (Fig 4D) some of these GS showed the presence of bilirubin also but with some metallic component, which could not be identified due to broadness of their ^13^C NMR spectra.

### Quantitative ^13^C NMR analysis

**Fig 5:**
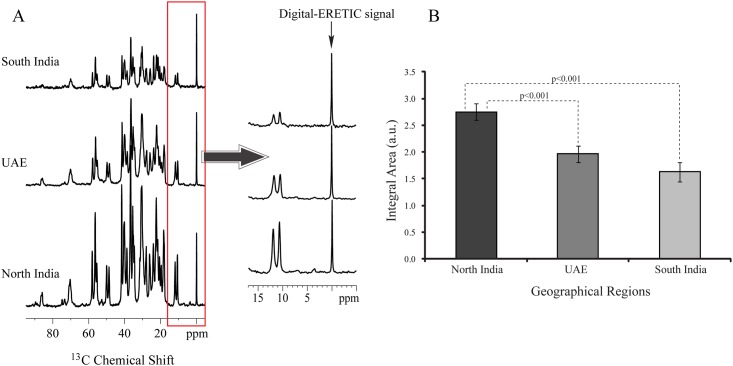
**(A) Showing digital-ERETIC signal at 0 ppm in solid-state** ^13^ C NMR **spectra for quantitation of cholesterol. (B) Showing the quantitation result of cholesterol for the three geographical regions**.

For quantifying organic components present in GS using solid state NMR spectroscopy, Digital ERETIC [16] method of quantitation has been used (Fig 5A). It was found that the cholesterol concentration varied in all three geographical regions (Fig 5B). From this analysis, it was clear that GS from North India contain very high quantity of cholesterol compare to South India and UAE GS. We have found significant value between North India and UAE (*p*<0.001), North India and South India (*p*<0.001), while comparison was not significant between UAE and South India (*p*>0.005).

### FTIR analysis of GS

**Fig 6:**
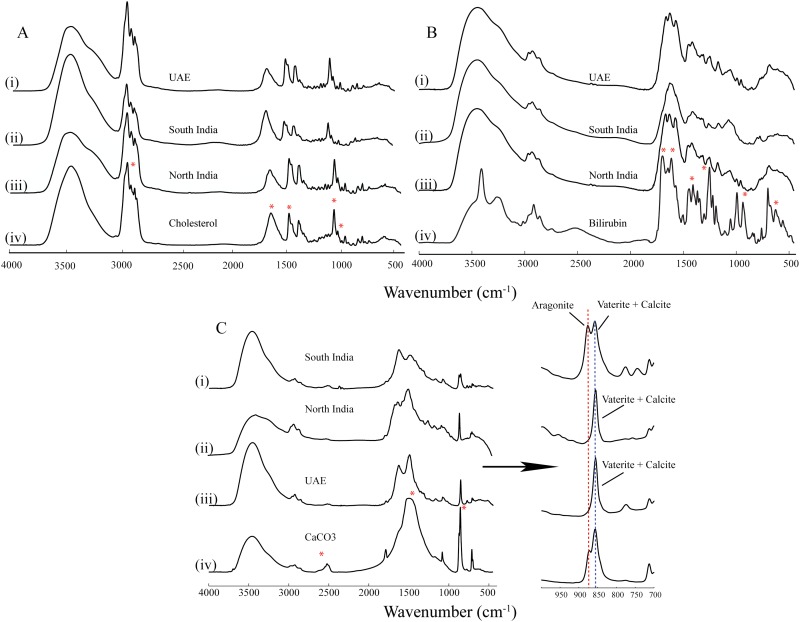
Showing presence of cholesterol, Bilirubin and CaCO_3_. In GS samples, compared with standards. Representative spectra from each region have been compared with the standard compound spectra showing the presence of reference compound in GS. Characteristic absorption peaks for cholesterol; bilirubin and CaCO3 are clearly visible in all GS spectra.

Overall 134 GS were subjected to FTIR for complete organic and inorganic constituent analysis. FTIR analysis of GS showed the presence of cholesterol, bilirubin, polymorphs of calcium carbonates, and some other compounds like peptides, mucin etc. (Fig 6). FTIR spectra of mixtures, CaCO_3_—cholesterol, CaCO_3_—bilirubin and bilirubin- cholesterol were used for unambiguous assignment of complex spectral regions of GS. Occurrence of CaCO_3_ was found in GS of all the three regions. Mg was not found in any GS. We have found very less number of pigmented (bilirubin) stones. Many GS samples belonging to all the three regions were black, harder and smaller in size but six UAE GS samples showed the bilirubin pattern. These stones were dark black in color and found to be harder than other region GS.

### Epidemiology of GS

GS are present in majority of patients with Gallbladder cancer (GBC) with occurrence of more than 90% in Chile, 60–70% in India and 50–60% in Japan. Patients with GS have 7 fold higher risk of GBC than normal population [17]. The risk of GBC in patients with GS is variable and is dependent on race and sex as well as on the duration of exposure to GS [18]. Risk of GBC has been reported to increase with increasing size of GS; patients with larger (>3 cm) GS have about 10 times higher risk of having GBC than those with smaller (<1 cm) size GS [19]. The numbers of GBC is higher in populations with high rate of GS prevalence. GS incidence in North India and UAE is prominent. Whereas GBC is rare in UAE and very frequent in North India [1]. The type of GS may also be important–>80% of GS in Northern India, where GBC is common, are cholesterol. GS from UAE also contain cholesterol as a major component. But we have found decreased content of cholesterol compared to North Indian GS. In Southern India, where GBC is less common, majority (>60%) of GS are pigment and only 5% are cholesterol GS. Apart from cholesterol and/ or bilirubin, calcium and magnesium salts are also present in GS. Madhulika *et al*. reported that calcium and magnesium concentrations were higher in patients having GS as compared to non-cancerous. The epidemiology of GS and GBC, both shows increasing incidences with age, preponderance in female grade and also governed by identical geographical and ethnic variations. Frequency of GS occurrence differs widely. A survey carried out in different geographical regions shows that the GS in Pima Indians (64–73%) in Arizona has highest prevalence in world. Canadian Indians (62%), Malpuche Indians (49%) and Mexican American (27%) also have higher frequency of GS. Proportionately, GS prevalence was lower in Northern Europe, Italy (14%), Norway (22%), Sweden (11–25%) and in Poland (20%). In Asian population, India (10–22%) has highest occurrence of GS while lower in China (5%), Japan (5%), Taiwan (5–12%), and Thailand (4%) [20]. In India, GS disease is higher in North, North east and east as compared to other regions of country and North Indian woman are more prone to having GS disease.

North India, South India and UAE have been well known for their great disparity and diversity in geographical location, culture and eating habits. The result of the study significantly shows that the population of GS disease of the area of high prevalence rate of both GS and GBC, found as a higher predominance of cholesterol level among them. The comparative results of the concentration of cholesterol in the area of high prevalence of GS and GBC was higher, and vice versa. Solid state NMR analysis revealed the presence of cholesterol as the major constituent as it was found among 98% North Indian GS patients; however other 2% these patients have bilirubin associated GS disease. However, in South Indian ~ 45% of population showed cholesterol associated stones and remaining 55% GS of the respective population were associated with bilirubin. In another study on UAE population, 75% of GS showed the presence of cholesterol as major constituent with and remaining 25% of population were bilirubin associated GS. Composition analysis of GS from regions with high and low prevalence and the prevailing incidence rates of GS are very important for understanding the pathogenesis of GS. Our data supports the fact that dietary habit could be the main cause of cholesterol, bilirubin and CaCO_3_ variation in all region as higher concentration of these components can increase deleterious effect on gallbladder and can alter the composition of bile, which ultimately may lead to GS formation [21]. There are many studies which emphasize the role of diet (energy intake, cholesterol, sugar, carbohydrate) as a potential risk factor for GS. Biliary phospholipid concentration plays an important role in cholesterol precipitation, which directly reflects in dietary habit, and can lead to the formation of gallstone. High intake of saturated fats and refined sugars may increase the risk of gallstone formation [22]. A widespread effect of dietary habits on GS could be seen by studying patients from different regions. Frequency of GS occurrence differs widely.

An X-ray diffraction study on GS from England and Australia revealed that the main constituent of stones is cholesterol monohydrate [14]. We have also found cholesterol as a main constituent with higher concentration in North Indian and UAE while lower in South India. Similar study from Northern Germany on a very large dataset comprising ~1000 of stones showed that cholesterol stones predominate in Germany, as cholesterol was present in ~95% GS, bilirubin was present in around 30% of stones in varying amounts and ~10% stones had different salts of calcium [23]. Studies from South India revealed similar results with cholesterol and bilirubin as main constituents. Similar results have been obtained when FTIR spectroscopy were used for the compositional analysis of GS [24, 25]. One such study from Israel employed these techniques to reveal predominance of bilirubin stones in its native geographical region [26]. We have also found bilirubin as a main constituent in South Indian GS.

## Conclusion

Compositional analysis of GS from different geographical regions can provide important insights in to the correlation between GS disease and GBC. These regions have different environmental factors and dietary habits, which plays an important role in the GS formation.

We have compared the compositional analysis of organic and inorganic components of GS from three different geographically regions with high/low prevalence and incidence rates of GS/GBC using solid state NMR and FTIR spectroscopic techniques. GS from regions with high prevalence/incidence rates of GS and GBC contained much higher concentration of organic component (cholesterol) and very less inorganic content (specifically CaCO_3_) as compared to other two regions with low incidence rates of GBC. Regions where prevalence rates of both GS disease and GBC are higher have relatively larger sized stones whereas GS from other regions are very small in size. Solid state NMR in conjunction with FTIR can provide a very good picture of organic and inorganic substances in GS.

## Supporting Information

S1 File Additional NMR spectrum are given as supporting file. Fig A in S1 File. ^13^C NMR Spectra of human GS from North India. GS containing cholesterol (1–8), GS containing inorganic solvents or some metallic compounds (9–10, generally black in colour). Fig B in S1 File. ^13^C NMR Spectra of human GS from South India. GS containing cholesterol (1–6), GS containing inorganic solvents or some metallic compounds (7–10, generally black in colour). Fig C in S1 File. ^13^C NMR Spectra of human GS from UAE. GS containing cholesterol (1–6), GS containing inorganic solvents or some metallic compounds (7–10, generally black in colour)
(DOCX)
