# Supplementary material for: Gallstones: A Worldwide Multifaceted Disease and Its Correlations with Gallbladder Carcinoma
Source: PLoS One. 2016 Nov 10;11(11):e0166351. doi: 10.1371/journal.pone.0166351 (PMC5104357; doi:10.1371/journal.pone.0166351)
Supplement: S1 File — Fig A in S1 File. 13C NMR Spectra of human GS from North India. GS containing cholesterol (1–8), GS containing inorganic solvents or some metallic compounds (9–10, generally black in colour). Fig B in S1 File. 13C NMR Spectra of human GS from South India. GS containing cholesterol (1–6), GS containing inorganic solvents or some metallic compounds (7–10, generally black in colour). Fig C in S1 File. 13C NMR Spectra of human GS from UAE. GS containing cholesterol (1–6), GS containing inorganic solvents or some metallic compounds (7–10, generally black in colour) (DOCX) [file pone.0166351.s001.docx]

Gallstones: a worldwide multifaceted disease and its correlations with gallbladder carcinoma

**Short title:** Analysis of gallstone using NMR and FTIR spectroscopy

Raj Kumar Sharma^1,2^, Kanchan Sonkar^1,3^, Neeraj Sinha^1,^ Rebala Pradeep^4^, Ahmad Ebrah Albani ^5^, Anu Behari^6^ , Duvvuri Nageshwar Reddy^4^, Alvina Farooqui^2^, Vinay Kumar Kapoor^6^

**Authors affiliations:**

1. Centre of Biomedical Research, Sanjay Gandhi Post Graduate Institute of Medical Sciences (SGPGIMS) - Campus, Lucknow, India
2. Department of Biosciences, Integral university, Kursi road, Lucknow, India
3. Department of Biochemistry & Biophysics University of Pennsylvania Perelman School of Medicine, Philadelphia, U.S.A
4. Department of Surgical Gastroenterology, Asian Institute of Gastroenterology, Somajiguda, Hyderabad, Andhra Pradesh, India
5. Department of Surgery, Zayad Military Hospital, Abu Dhabi, UAE
6. Department of Surgical Gastroenterology, SGPGIMS, Raibarelly Road Lucknow, Uttar Pradesh, India

***Correspondence to:**

Dr. Neeraj Sinha

Centre of Biomedical Research, Sanjay Gandhi Postgraduate Institute of Medical Sciences Campus, Raibareli Road, Lucknow-226014 (INDIA)

E-mail: [neerajcbmr@gmail.com](mailto:neerajcbmr@gmail.com), [neeraj.sinha@cbmr.res.in](mailto:neeraj.sinha@cbmr.res.in)

Contact: +91-0522-2495034

Fax: +91-522 – 2668215

Supporting Information (SI)


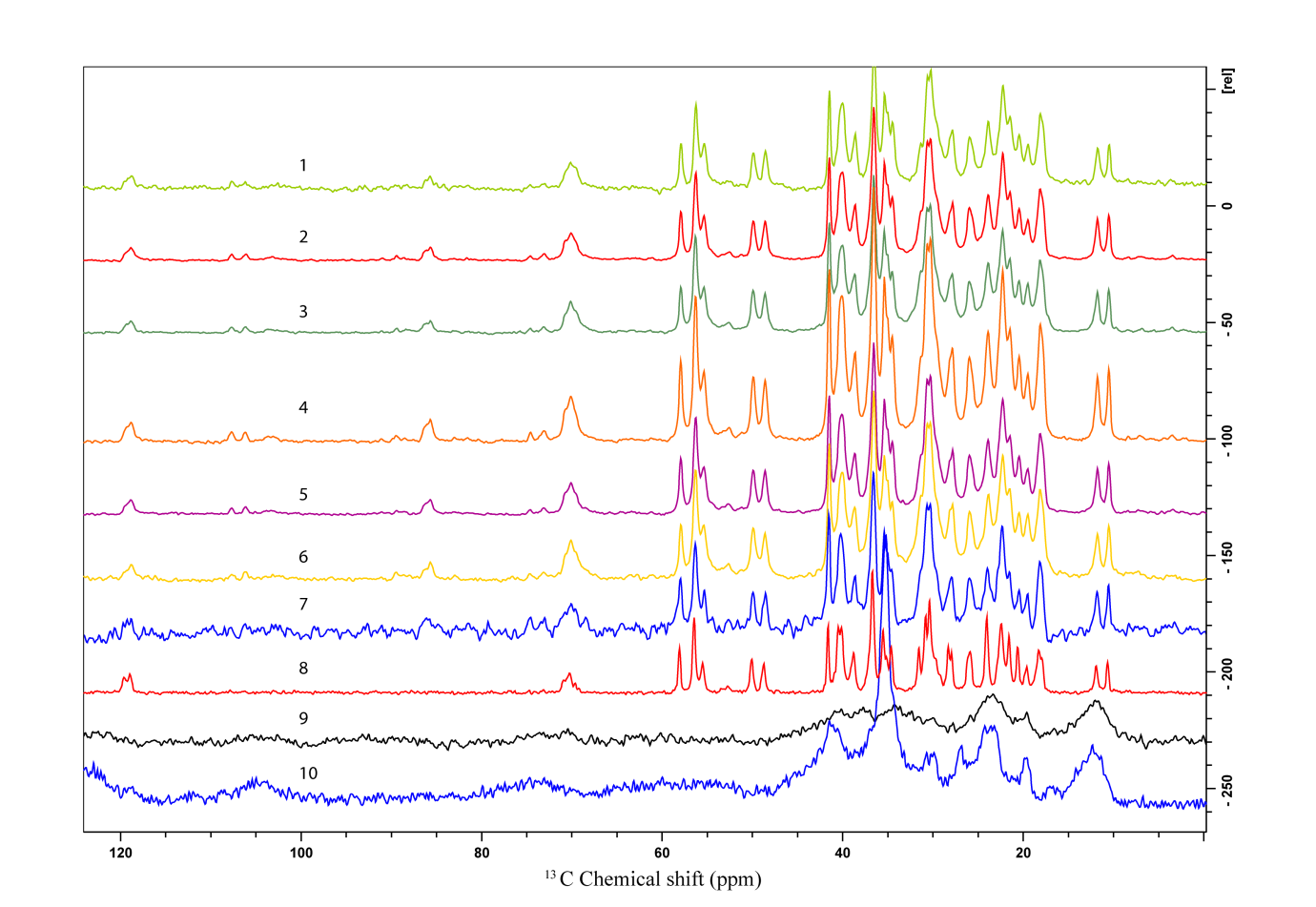


Fig. A. ^13^C NMR Spectra of human GS from North India. GS containing cholesterol (1-8), GS containing inorganic solvents or some metallic compounds (8-10, generally black in colour)


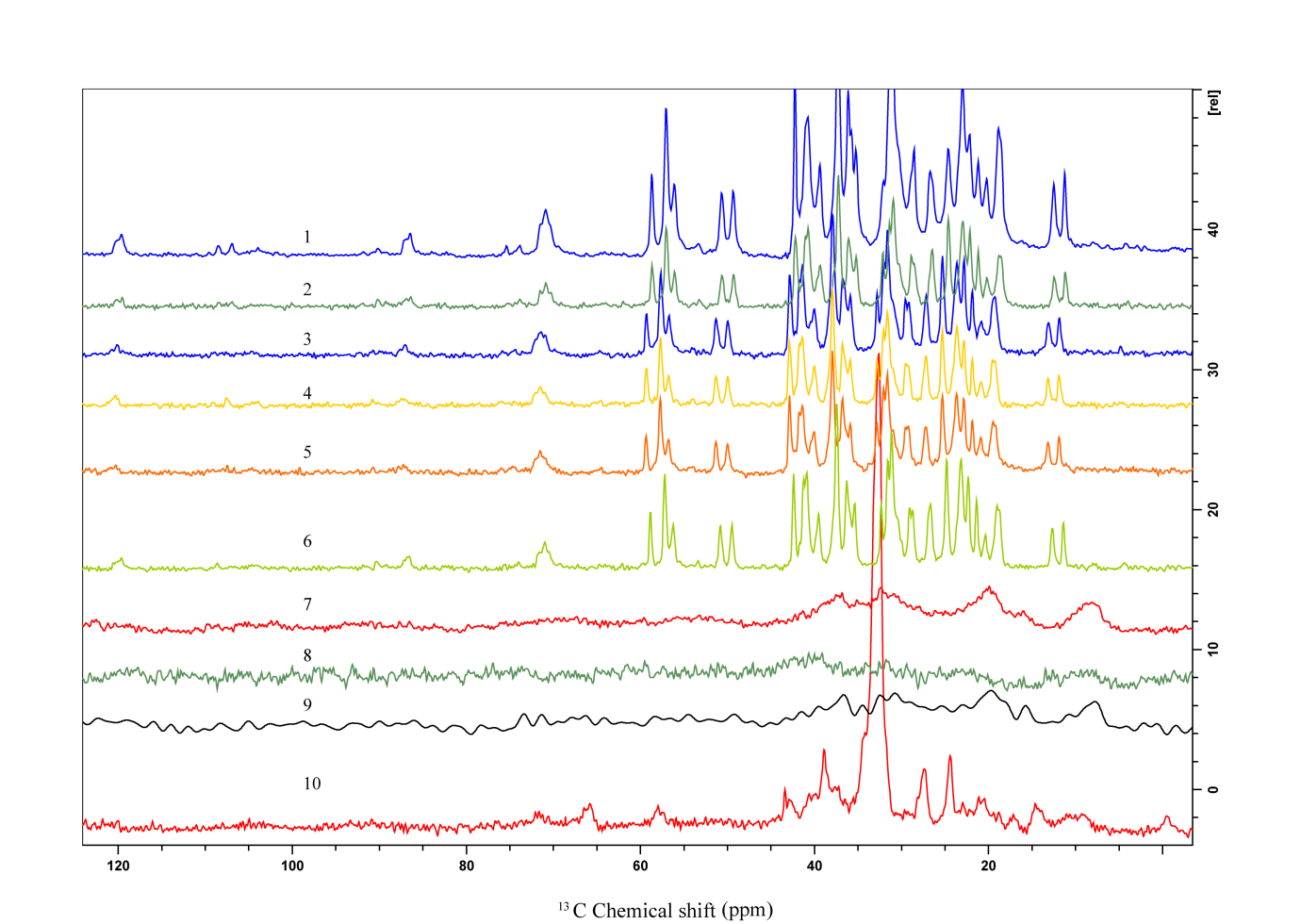


Fig. B. ^13^C NMR Spectra of human GS from South India. GS containing cholesterol (1-6), GS containing inorganic solvents or some metallic compounds (7-10, generally black in colour)


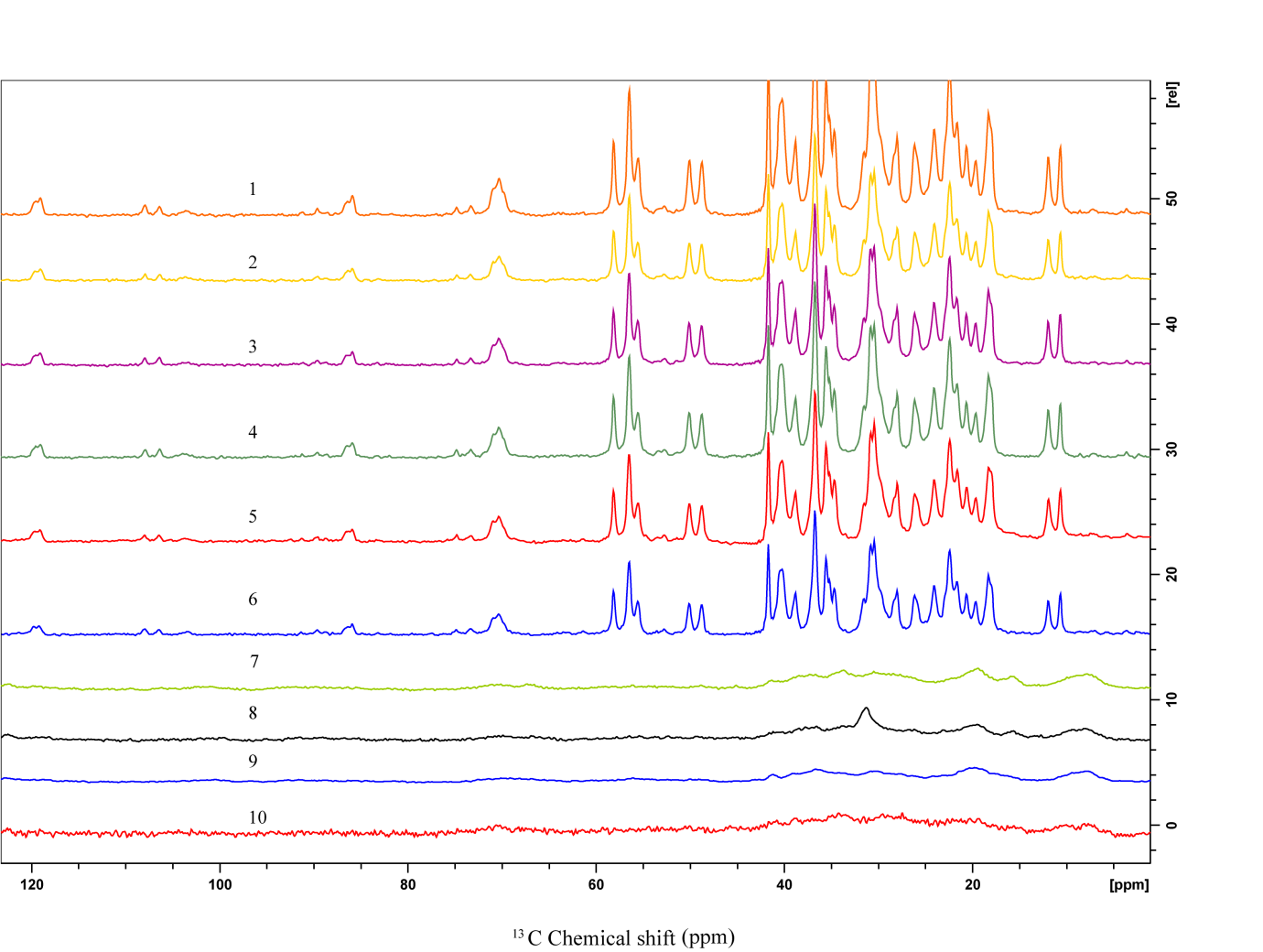


Fig. C. ^13^C NMR Spectra of human GS from UAE. GS containing cholesterol (1-6), GS containing inorganic solvents or some metallic compounds (7-10, generally black in colour)
